# Hybrid Materials Based on Self-Assembled Block Copolymers and Magnetic Nanoparticles—A Review

**DOI:** 10.3390/polym17101292

**Published:** 2025-05-08

**Authors:** Galder Kortaberria

**Affiliations:** Group ‘Materials + Technologies’ (GMT), Chemical and Environmental Engineering Department, Faculty of Engineering of Gipuzkoa, University of the Basque Country (UPV/EHU), Plaza Europa 1, 20018 Donostia-San Sebastian, Spain; galder.cortaberria@ehu.eus

**Keywords:** magnetic nanoparticles, block copolymers, nanocomposites, templates, patterns

## Abstract

In this review work, the different routes and methods for preparing hybrid materials based on nanostructured block copolymers (BCPs) and magnetic nanoparticles (MNPs) are analyzed, as they can be potentially employed in different sectors like biomedicine, electronic or optoelectronic devices, data storing devices, etc. The first procedure for their preparation consists of the nanostructuring of BCPs in the presence of previously synthesized NPs by modifying their surface for increasing compatibility with the matrix or employing magnetic fields for NP orientation, which can also promote the orientation of nanodomains. Surface modification with surfactants led to the selective confinement of NPs depending on the interaction (mainly hydrogen bonding) degree and their intensity. Surface modification with brushes can be performed by three methods, including grafting from, grafting to, or grafting through. Those methods are compared in terms of success for the positioning and confinement of NPs in the desired domains, showing the crucial importance of brush length and grafting density, as well as of NP amount and modification degree in the self-assembled morphology. Regarding the use of external magnetic fields, the importance of relative amounts of MNPs and BCPs employed and that of the magnetic field intensity for the orientation of the NPs and the nearby BCP domains is shown. The second procedure, consisting of the in situ synthesis of NPs inside the nanodomains by a reduction in the respective metallic ions or employing metal-containing BCPs for the generation of MNP patterns or arrays, is also shown. In all cases, the transference of magnetic properties to the nanocomposite was successful. Finally, a brief summary of some aspects about the use of BCPs for the synthesis, encapsulation, and release of MNPs is shown, as they present potential biomedical applications such as cancer treatment, among others.

## 1. Introduction

Organic/inorganic nanocomposites are hybrid materials in which an inorganic filler or nanoparticles (NPs) are dispersed or selectively placed into the organic part, usually a polymeric matrix. Those inorganic fillers or NPs transfer their specific optical, electric, or magnetic properties to the hybrid material [1,2,3], with potential applications in different sectors such as solar cells, sensors, or magnetic storage devices, among others [4,5,6]. Some of the most important hybrid materials are those in which block copolymers (BCPs) constitute the organic part.

BCPs can self-assemble, generating nanostructures such as lamellar, cylindrical, or spherical ones, among others. For this reason, they can be employed to lead to the self-assembly of NPs inside those nanostructures, resulting in materials designed at the nanoscale that can present improved properties [7]. BCPs can be employed either for the synthesis of NPs or for their placement in high-ordered nanostructures, resulting in nanomaterials with specific optical, magnetic, or electronic properties. The concept of synergistic assembly should be mentioned at this point, as BCPs can rearrange their nanostructures due to their interactions with the inorganic fillers.

Many works can be found in the literature related to hybrid materials based on several BCP matrices and different inorganic NPs, such as silver [4,8], gold [9,10], TiO_2_ [1,11], or quantum dots like CdSe [3,12,13,14], among others, but there is still work ongoing and to be conducted in this field, and is the object of many investigations.

On the other hand, magnetic nanoparticles (MNPs) have been deeply investigated by many researchers in many fields, such as magnetic fluids [15], catalysis [16], biotechnology [17], magnetic resonance imaging [18], and data storage [19]. Different types of MNPs have been synthesized with different compositions and phases, including iron oxides [20]; pure metals such as Fe and Co [21,22]; spinel-type ferromagnets such as MgFe_2_O_4_, MnFe_2_O_4_, and CoFe_2_O_4_ [23,24]; and alloys such as CoPt_3_ and FePt [25,26]. During the last few years, several investigations have been conducted on the synthesis of iron oxides such as Fe_3_O_4_ (magnetite), α-Fe_2_O_3_ (hematite), γ-Fe_2_O_3_ (maghemite), FeO (wüstite), ε-Fe_2_O_3_, and β-Fe_2_O_3_ [27], with magnetite and maghemite being the most employed ones.

Regarding the procedures employed for the fabrication of hybrid BCP–MNP nanocomposites, they can be divided into two main types. The first one consists of first synthesizing the NPs with a controlled size (or using commercial ones), and then nanostructuring the BCP in their presence, taking into account that the presence of NPs can modify matrix morphologies due to interactions or by selective positioning. The second procedure consists of the in situ synthesis of MNPs inside the nanostructured BCP during the nanostructure formation in controlled conditions.

For the first procedure, several strategies can be employed. One of them is the modification of the MNP surface with surfactants or with polymeric chains (brushes) grown on their surface. The coating of the NP surface with surfactants leads to interactions (hydrogen bonding, hydrophilic or hydrophobic attraction) with a specific block of the BCP in order to be placed at those domains, in addition to avoiding the formation of aggregates, which is a trend with most of NPs [28,29,30,31]. This is the easiest procedure, as there are many surfactants available and the chemistry is relatively simple. Another strategy is the growing of polymeric chains (brushes) that present affinity (or even are the same) with one of the blocks for the selective placement of MNPs at the desired domains [3,12,13,32,33,34,35]. There are three different methods for brush growing, known as grafting to, grafting from, and grafting through. All those procedures will be explained and compared in their respective sections of the manuscript, including a table for a better understanding.

The last of the strategies for the first main procedure is the employment of magnetic fields for the alignment or selective placement of MNPs in nanostructured BCPs [29,31,36,37]. In this case, a magnetic field whose intensity is controlled is applied during nanocomposite formation. Several authors showed that, by controlling the intensity (usually with the distance between magnets) of the magnetic field and the amount of MNPs employed, alignment can be achieved, in some cases orienting the BCP domains surrounding the MNPs. 

**Figure 1:**
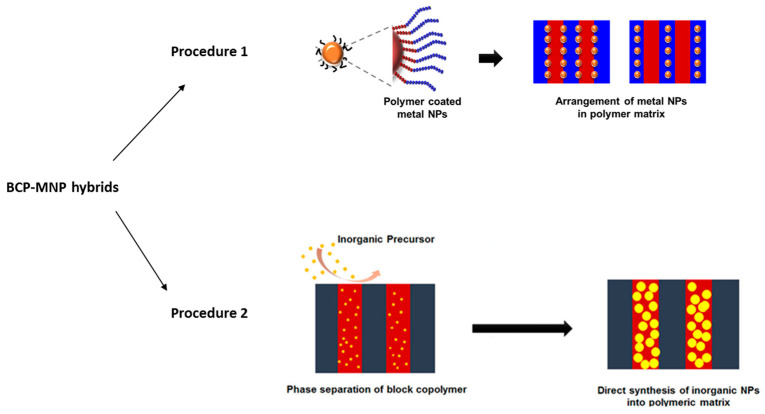
Schematic representation of the two main procedures for the preparation of BCP–MNP hybrid materials. (Adapted with permission from [48], Frontiers 2015).

The second main pathway employed for hybrid nanocomposites consists of synthesizing MNPs in situ, inside the nanodomains corresponding to one of the blocks, by a reduction in the corresponding metallic ions. In this vein, during the last few years, there has been ongoing research on the employment of BCPs as templates for the synthesis of MNP arrays with specific locations, or even for MNP encapsulation and posterior release, with potential applications in biomedicine or drug delivery [38,39,40,41,42,43,44,45]. The scheme for both main pathways can be seen in Figure 1.

Examples of the proper dispersion and placement of MNPs without any modification or magnetic field can also be found [46], as well as of the control of particle/solvent and polymer/solvent interactions in order to promote the selective placement of MNPs at the desired domains of the block copolymer [47]. 

In the present work, the studies performed by many authors on the synthesis and characterization of BCP/MNP hybrid systems by different strategies or methods are reviewed, showing the main findings and potential applications of the generated materials. The review will be divided into sections regarding the different strategies employed by authors: surface modification with surfactants, the growing of polymer brushes, the use of the magnetic field, and the role of BCPs as templates for MNP synthesis, encapsulation, or release. This last part will only be introduced and shown briefly, as there are many ongoing studies, and the aim of the present review is mostly to show the rest of the procedures for preparing thin-film composites based on hybrid BCP/MNP materials.

## 2. Surface Modification with Surfactants

This method, considered the simplest one, is based on the use of previously synthesized MNPs, with the surface modified with surfactants, which are added to the BCP that is afterwards assembled into different nanostructures [28,29,30,31]. Surfactant molecules that coated the surface of the MNPs present higher affinity towards one of the blocks, leading to the confinement of the MNPs into the nanodomains corresponding to that block. However, several authors have pointed out that, especially for ferromagnetic nanofillers such as iron oxides, cobalt, and nickel, the strong magnetic attractions among them led to the aggregation of MNPs.

Several studies can be found on the use of surfactants for dispersing MNPs in nanostructured BCPs. Char et al. [28] employed poly(styrene-b-isoprene) (PS-b-PI) assembled into PI cylinders and poly(styrene-b-4-vinylpiridine) (PS-b-P4VP) assembled into PS cylinders as templates for the placement of oleic acid-coated Fe_2_O_3_ MNPs. Their main finding was that, independently of the employed BCP, the placement of MNPs and the formation of aggregates depended on the solvents employed for casting nanocomposite films: When solvents solving both blocks were employed, the aggregation of nanofillers occurred during film formation. On the other hand, with the use of solvents selective for one of the blocks (forming the cylinders), MNPs were selectively placed into the cylinders and the inversion of micelles occurred during film formation. They concluded that depending on the degree of interaction between the MNPs and one of the blocks, nanofillers should be placed either at the interfaces (disrupting the nanostructure when the MNPs were increased) or inside the minor domains. 

Park et al. [31] also employed oleic acid-capped Fe_2_O_3_ nanoparticles for dispersal into a poly(3-hexylthiopene-b-ethylene glycol) (P3HT-b-PEG) copolymer by a magnetic field. For this reason, this study will be analyzed in the section devoted to magnetic field application.

Miles et al. [30] employed the hydrogen bonding interactions among gallic acid-capped FePt nanoparticles and P2VP nanodomains of a PS-b-P2VP diblock copolymer to selectively place them into those nanodomains, obtaining materials with good magneto-optical properties and presenting little trade-off in terms of scattering loss. MNPs improved the Faraday rotation properties. The control of BCP nanostructures and surface functionalization of MNPs allowed for hybrid materials with potential magneto-optical applications to be obtained. 

**Figure 2:**
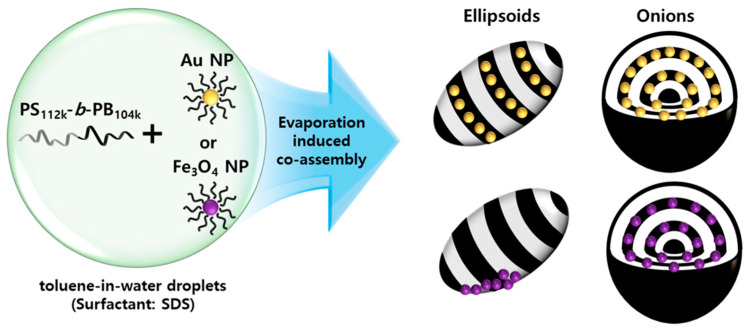
Illustration showing co-assembly of inorganic nanoparticles (Au NP and Fe_3_O_4_ NPs) with PS-b-PB copolymer by controlled solvent evaporation from a toluene-in-water emulsion. Reproduced with permission from Ref. [49].

J. Shin et al. [49] analyzed the effect of MNPs on the nanostructure of nanocomposites formed by symmetric poly(styrene-b-1,4-butadiene) (PS-b-PB) BCP with Fe_3_O_4_ or Au nanoparticles, comparing the effect of NP type as well as their placement at the nanodomains. They first synthesized Au and Fe_3_O_4_ nanoparticles stabilized with oleyl ligands. The confined co-assembly of symmetric poly(styrene-b-1,4-butadiene) (PS-b-PB) BCP and NPs in evaporative emulsions resulted in particles with various morphologies, including striped ellipsoids, onion-like particles, and their intermediates. The main difference among PS-b-PB/Au and PS-b-PB/Fe3O4 was in the distribution of NPs, which affected the overall particle morphology. In the case of PS-b-PB/Fe_3_O_4_ ellipsoids, MNPs clustered and segregated to the particle/surrounding interface of the ellipsoids, being selectively placed in the middle of the PB domains in a string-like pattern for PS-b-PB/Fe_3_O_4_ onion-like particles. The scheme of the employed procedure is shown in Figure 2.

## 3. Surface Modification with Polymeric Brushes

Polymer brushes are polymeric chains anchored or grafted to a surface, with a grafting density high enough for the surface-attached chains to become crowded and expand outward from the surface [50]. Depending on the employed criteria, different classifications of brushes can be found. Depending on the use of linear chains, planar surfaces, or spherical NPs as a substrate for brush growing, brushes can be divided into one-dimensional, two-dimensional, or three-dimensional, respectively. If their composition and architecture are taken into account, brushes can be classified into homopolymeric, mixed homopolymeric, block copolymer, or branched polymer brushes. 

**Figure 3:**
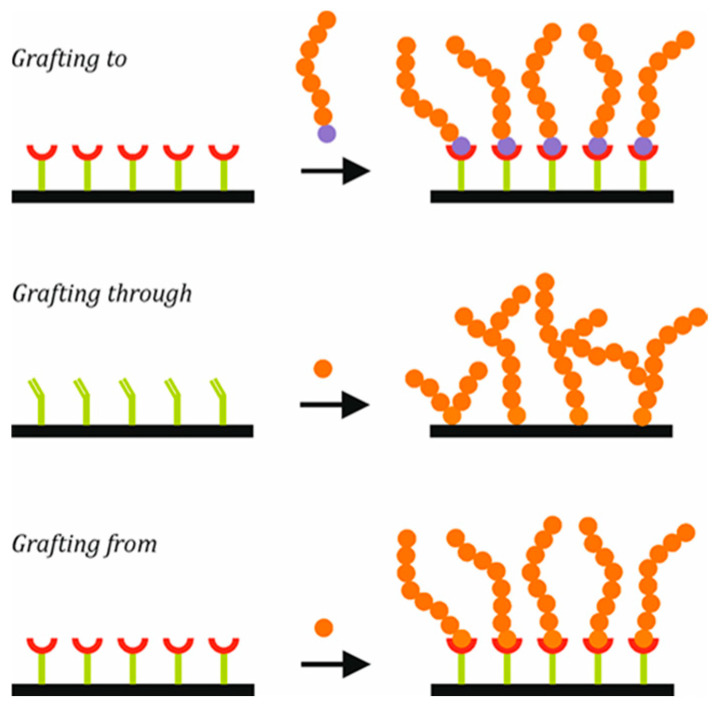
Different techniques for polymer brush grafting.

Moreover, different methods can be used for grafting polymeric chains to NP surfaces, as shown in Figure 3. 

The first method is known as grafting to: polymers with suitable end-functional groups react with appropriate surface sites on the inorganic nanoparticles [51,52]. The second method, grafting through, consists of linking a monomer by a covalent bond to a surface, from which a surface copolymerization is carried out, incorporating the inorganic part in the polymer chains [13,53]. The growth of chains is initiated in solution, and during propagation, a surface-bound monomer unit can be integrated into the growing chains, with polymer chains being permanently grafted to the surface. The growth of chains can continue with the addition of new units that are either free or attached to the surface. The third method, known as grafting from, consists of grafting initiator molecules (or surfactant molecules) to the surface, from which chains will grow in situ. Several techniques can be used for the polymerization: controlled radical polymerization (CRP), ionic living polymerization, or ring-opening polymerization (ROP) [54,55].

Many works can be found in the literature regarding the use of polymeric brushes for increasing the compatibility with a BCP matrix and placing the MNPs into the desired domains. Those works will be classified in terms of the method employed for the generation of brushes.

### 3.1. Brushes by the Grafting from Method

Initiator molecules previously anchored to the surface of NPs are responsible for the chain growth, which can occur via different polymerization techniques, obtaining high grafting densities as well as more finely tuned control of the molar mass and polydispersity. Among the variety of radical polymerization methods that can be used (atom transfer radical polymerization (ATRP), reversible addition–fragmentation chain transfer, and nitroxide mediated polymerization), ATRP has been the most widely employed by different authors. In order to obtain uniform growth of chains, the step consisting of an atom transfer is the most important, for which two techniques can be used: the use of a sacrificial free initiator or the employment of a persistent radical for better control of the polymerization.

I. Garcia et al. [56,57,58,59] grafted PS or PMMA brushes from the surface of Fe_3_O_4_ MNPs by the ATRP technique in order to selectively confine them into PS or PMMA nanodomains of different self-assembled BCPs. Firstly, they grafted PS brushes from an NP surface (PS–MN) to add them (1, 2, and 4 wt% NPs) to a commercial poly(styrene-b-butadiene-b-styrene) (SBS) block copolymer [56,57]. A grafting density of 0.9 chains/nm^2^ was obtained, in addition to measuring the molar mass of the growth chains after cleavage. After solution mixing, the mixtures were cast onto silicon wafers, nanostructuring them by thermal annealing. By measuring the Tg of both blocks from the BCP and analyzing the effect of MNPs on them (both modified and unmodified ones) and characterizing the obtained morphologies in terms of atomic force microscopy (AFM), they pointed out that PS–MN was located at the PS nanodomains. The improvement in mechanical properties observed for the SBS/PS–MN system compared to that of the SBS/MN one indicated higher interactions and better interfacial adhesion promoted by the PS brushes. Moreover, the increase in the thickness of the PS lamellae, disrupting the morphology, indicated the presence of PS–MN at PS domains. In conclusion, they pointed out that a very good and selective dispersion of PS–MN was obtained in the SBS, maintaining the morphology of a neat copolymer and increasing the mechanical properties.

In a second study, they grafted PMMA brushes (with a grafting density of around 0.1 chains/nm^2^) from the surface of magnetic Fe_3_O_4_ nanoparticles (PMMA–MN) by ATRP to prepare nanocomposites with a poly(2-vinylpyridine-b-methylmethacrylate) (P2VP-b-PMMA) block copolymer by solution mixing and spin coating onto glass wafers [58]. The obtained films were annealed by exposure to CCl_4_ vapors (selective for PMMA)—that is, by solvent vapor annealing (SVA). They employed both AFM and magnetic force microscopy (MFM) to analyze the effect of SVA and MNP modification on generated nanostructures or morphologies. By analyzing the effect of exposure time on the morphology of the neat BCP, they found an evolution of the nanostructure: While a hexagonal morphology was obtained after 3 h of exposure, after 9 h, a quasi-lamellar morphology normal to the substrate was observed (as CCl_4_ is selective for PMMA, it promoted the migration of P2VP from the free surface to the substrate). After 14 h, PMMA migrated to the surface, forming the continuous phase, while P2VP appeared as the discontinuous one. They obtained several conclusions by analyzing the nanocomposites: For 2 wt% PMMA–MN, the hexagonal nanostructure of the neat BCP was maintained, with PMMA–MN being selectively placed at PMMA domains. On the other hand, for the nanocomposite with 4 wt% PMMA–MN, they observed that some agglomerations began to appear. Moreover, the transference of the magnetic properties of the NPs to the nanocomposite thin films was confirmed by MFM. 

C. Xu et al. [33] also employed the ATRP polymerization technique for grafting PMMA brushes from the surface of Fe_3_O_4_ MNPs with the aim of obtaining a good dispersion into a nanostructured PS-b-PMMA block copolymer. Brushes with 2.7, 13.3, and 35.7 kg/mol were obtained, with a grafting density of 0.73 chains/nm^2^. Mixtures (with 1 to 16 wt% MNPs) prepared in toluene were spin coated onto silicon wafers to then be thermally annealed for different times at 160 °C. The increase in the molecular weight of the brushes led to a poorer dispersion of MNPs, which tended to form aggregates. They found two types of aggregates: small ones, located at the defects of the lamellae, and larger ones that disrupted the nanostructure, appearing to be encapsulated by onion-ring copolymer structures. The parallel lamellar nanostructure of the neat BCP was maintained for the nanocomposite containing 1 wt% NPs modified with brushes presenting the lowest molar mass, with the nanoparticles appearing to be properly dispersed into the PMMA domains. The higher amount of those NPs avoided the constitution of lamellar morphology, as they tended to aggregate. When NPs grafted with 13.3 kg/mol brushes were employed for nanocomposites, films with 4 wt% NPs presented small aggregates within the PMMA domains, as well as some big agglomerates placed at the boundaries that were unable to be confined into the PMMA domains. For all nanocomposites with NPs presenting brushes of 35.7 kg/mol, aggregates were formed independently of the amount, disrupting the morphology, as their size appeared to be bigger than the lamellar period. The importance of the molar mass of the brushes grafted to the NPs was shown.

S. Douadi-Masrouki et al. [34] also grafted PS brushes from a magnetic γ-Fe_2_O_3_ nanoparticle surface by ATRP in order to incorporate them into lamellar poly(styrene-b-butyl methacrylate) (PS-b-PBMA) diblock copolymer films. They were synthesized by ATRP symmetrical diblock copolymers in order to obtain lamellar morphologies. Nanocomposites were prepared by solution mixing in toluene, followed by spin coating onto silicon wafers and thermal annealing at 150 °C (well above the T_g_ of PS and below the order–disorder transition temperature) for 48 h. The neat BCP presented a lamellar nanostructure, with some defects on top of the films. For the NPs, they found that during the synthesis of the brushes, as a consequence of reactions among chains growing from adjacent NPs, they tended to form clusters. However, as the size of those clusters was lower than the lamella thickness, they were flattened between the lamellae, being compatible with self-assembly. They showed nanocomposites presenting the lamellar structure of the neat BCP, with NPs located in the lamellae, despite the NP dispersal being irregular.

**Figure 4:**
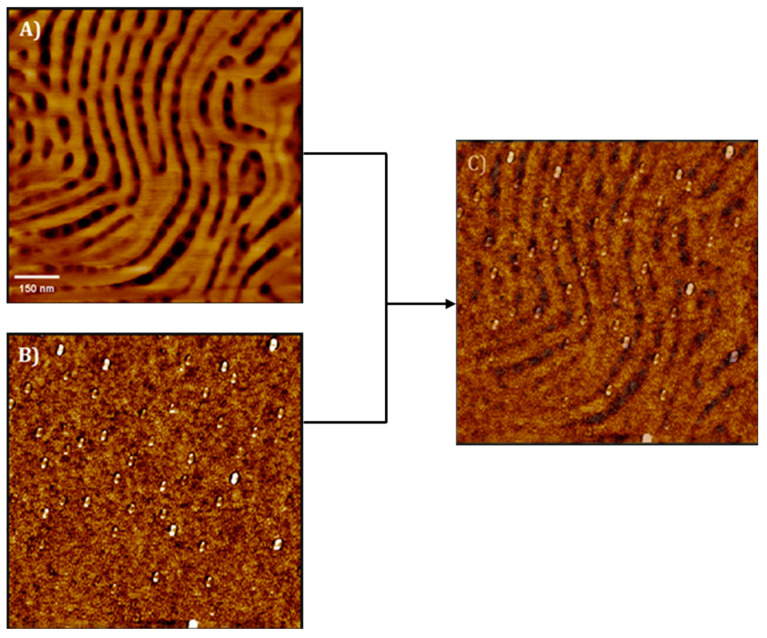
(**A**) AFM-phase image of the as-cast nanocomposite films with 1 wt% nanoparticles, (**B**) AFM-phase image of nanocomposite film with 1 wt% nanoparticles exposed to UV light irradiation for 48 h, and (**C**) superposition of (**A**,**B**). (Reproduced with permission from Ref. [60], copyright Elsevier 2016).

Barandiaran et al. [60] also functionalized Fe_2_O_3_ MNPs with PMMA brushes by ATRP and the grafting from method to prepare nanocomposites with a poly(isoprene-b-methylmethacrylate) (PI-b-PMMA) diblock copolymer. AFM and small-angle X-ray scattering (SAXS) were employed for the characterization of the obtained nanostructures, both for the neat BCP and for the nanocomposites. Morphological characterization of the neat BCP and the nanocomposites was performed in terms of AFM, both for the as-cast films and for those annealed with solvent vapors. The surface perpendicular lamellar nanostructure presented by the as-cast film of the neat BCP was changed to a mixed perpendicular and parallel lamellar morphology for a low NP concentration, with some cylinders appearing for higher NP content. When annealed under solvent vapors, the neat BCP was raised into a cylindrical morphology and maintained for nanocomposites with low NP concentrations. The highest NP concentration, however, led to the disruption of the morphology. By removing the organic part of the nanocomposites by UV irradiation, the authors confirmed the selective placement of the NPs into PMMA domains as a consequence of the presence of PMMA brushes on their surface, as shown in Figure 4. The transference of magnetic properties to the nanocomposites was corroborated in terms of magnetic characterization.

**Figure 5:**
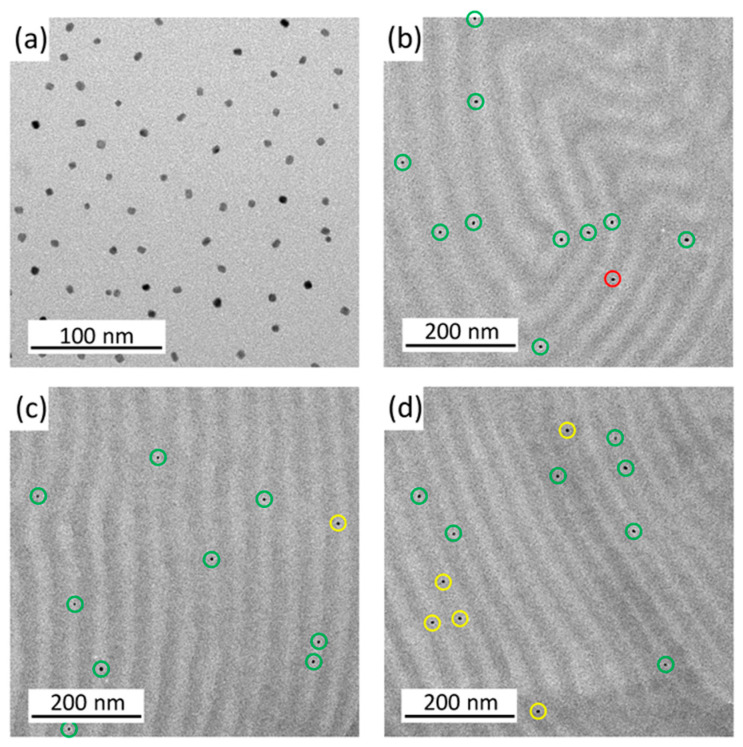
TEM images of the BCP films with 0.1 wt% FePt NP. (**a**) shows the pure FePt NPs, while (**b**–**d**) are different images of the lamellar PS-b-PMMA morphology with 0.1 wt% FePt NPs. The particles are marked with a green ring in the light PMMA phase, red in the dark PS phase, and yellow on the boundary [32].

Hartmann et al. [32] analyzed the effect of FePt NPs presenting PMMA brushes grafted from their surface on the nanostructures generated by the self-assembly of a PS-b-PMMa diblock copolymer. They analyzed the placement of NPs within PMMA domains at low NP amounts statistically, concluding that higher NP amounts altered the lamellar nanostructure of the neat BCP. The positioning of NPs into PMMA domains was also analyzed theoretically, and it was found that most of them were placed at lamellar PMMA domains. They concluded that, as the diameter of NPs is smaller than the width of PMMA domains, they did not alter the structure of the BCP morphology, as shown in Figure 5. For NP loadings of higher than 0.5 wt%, they negatively affected the order of the lamellar nanostructure. They demonstrated that the magnetic nature of the NPs inside the nanostructured morphology was maintained with bimodal magnetic force microscopy.

Upadhyaya et al. [35] employed Fe_3_O_4_ MNPs coated with PMMA brushes (grafting from technique) to prepare mixed-matrix membranes based on a poly(methacrylic acid-b-methyl methacrylate) (PMAA-b-PMMA) block copolymer synthesized by RAFT polymerization. They prepared thin-film membranes by casting or spin coating solutions prepared with different amounts of BCP in tetrahydrofurane and MNPs in water. The pore density was increased by the incorporation of MNPs in all cases, while for the film preparation method, those obtained by casting showed lower flux values compared to those prepared by spin coating, mainly because of the difference in membrane thickness obtained by each procedure. By applying an external magnetic film, the flux values of the membranes prepared by both methods were increased, probably due to the movement of the MNPs inside the organic matrix.

### 3.2. Brushes by Grafting to Method

The method consists of the employment of polymers with end-functional groups such as thiol groups, which are grafted to NP surfaces by the functional groups (such as –OH ones, among others) present on them. Though this method is not as extended as the previous one, several authors have employed it, mainly for modifying Au nanoparticles with polymeric brushes for selective placement in BCP nanodomains [61,62,63,64], but an interesting study with magnetic nanoparticles can also be found [65].

By this method, I. Barandiaran et al. [65] grafted a poly(methyl methacrylate-b-ε-caprolactone) (PMMA-b-PCl) diblock copolymer to the surface of Fe_2_O_3_ MNPs to prepare thin-film nanocomposites with poly(styrene-b-ε-caprolactone) (PS-b-PCl). They employed the ATRP polymerization technique to prepare a PMMA-b-PCl copolymer (21,000 g/mol) presenting terminal chlorine groups. MNPs were silanized with 3-aminopropyltryethoxysilane (APTS). 

**Figure 6:**
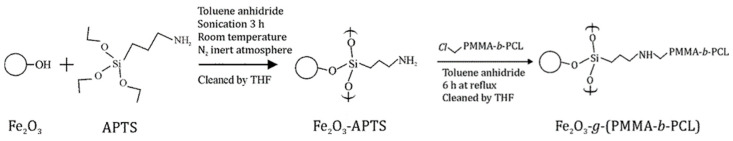
Reaction scheme for the APTS silanization reaction and PMMA-b-PCL copolymer anchoring by the grafting to reaction. (Reproduced with permission from Ref. [65], copyright Elsevier 2014).

BCPs were attached to the surface of MNPs by the grafting to method. The amine functionalities at the surface of the MNPs underwent an alkylation reaction with the terminal chlorine group, obtaining grafting density values of around 0.04 chains/nm^2^. The scheme for both procedures is shown in Figure 6.

Nanocomposite thin films containing 2 and 5 wt% MNPs were prepared by spin coating BCP/MNP solutions in toluene glass substrates. The films were thermally annealed under vacuum at 100 or 120 °C. The neat BCP self-assembled into a worm-like nanostructure for the films annealed at 100 °C, while a lamellar morphology was obtained for those annealed at 120 °C. Nanocomposites resulted in the same morphologies as those of the neat BCP, both at 100 and at 120 °C. By comparing nanocomposites with modified and unmodified MNPs, they found that those with brushes were better dispersed. The use of unmodified MNPs resulted in aggregates, altering the morphology of the BCP. The presence of PCL blocks at the surface seemed to improve the compatibility with the BCP matrix. Regarding the selective placement of MNPs, they claimed that even though some of them appeared at PCL domains, most of them were placed at the interface between the PCL and PS domains, without altering the BCP nanostructure. For samples annealed at 100 °C, MNPs presented average sizes of 25 and 40 nm (for nanocomposites with 2 and 5 wt%, respectively), while for films annealed at 120 °C, average sizes of 22 and 38 nm were measured. As the initial size of the MNPs was around 9 nm, they concluded that the MNPs were not individually dispersed. By TEM characterization they showed that during the modification procedure, the MNPs formed agglomerates surrounded by PMMA-b-PCL, presenting average sizes that were very similar to those found in the nanocomposites. Thus, aggregates were formed during the modification process and not during their dispersion into the BCP. The modification of the surface improved the MNP dispersion without altering the nanostructure of the BCP, as was previously found by other authors. 

### 3.3. Brushes by Grafting Through Method

By this method, molecules presenting functional groups that are able to polymerize (silanes with vinyl groups, for example) are fixed to the surface of MNPs. In this way, NPs present in the polymerization medium are covered by the polymer. Usually, this method results in higher grafting densities compared to the grafting to technique, besides being relatively easier to perform. A kind of network is formed as polymer chains at the surface present bonds among them, due to the multifunctionality of the MNP surface [66].

Following a procedure from Etxeberria et al. [13] employed for grafting brushes to self-synthesized CdSe quantum dots, some authors from the same group used this method to generate PS or PMMA brushes at the surface of Fe_2_O_3_ MNPs, to then be dispersed into different BCPs [67,68,69]. 

**Figure 7:**
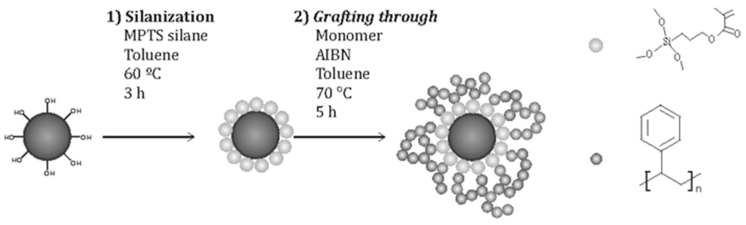
Scheme of the grafting through procedure for NP modification.

In their first work, Barandiaran et al. [67], functionalized the surface of Fe_2_O_3_ MNPs with PS brushes by this technique (Figure 7) to prepare nanocomposites with PS-b-P4VP. 

**Figure 8:**
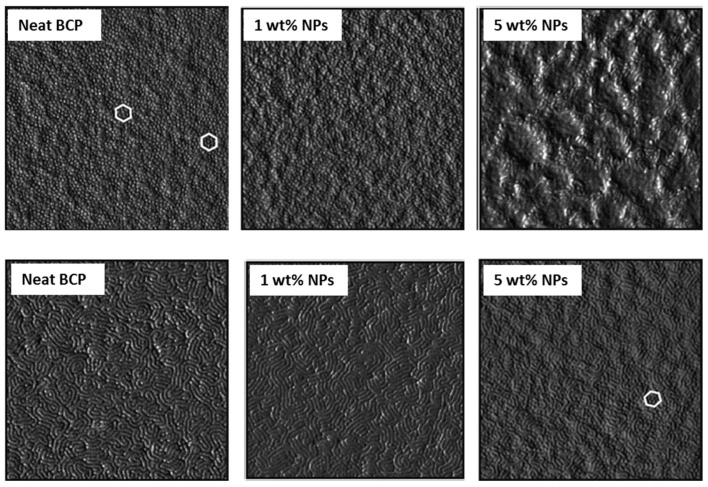
AFM-phase images showing the morphological evolution of the neat BCP and nanocomposites after 24 h (upper images) and 48 h (lower images) of exposure to dioxane vapors.

The amount of MNPs varied between 1 and 5 wt% at the nanocomposites. Thin films were annealed with saturated dioxane (selective for the PS block) vapors for several exposure times. When samples were annealed for 24 h, the hexagonal morphology of the neat BCP was maintained for the nanocomposite with 1 wt% NPs. However, by increasing the MNP amount, the morphology of the BCP was disrupted. When the films were annealed for 48 h, the neat BCP self-assembled into a stripped morphology, presenting a lamellar structure normal to the substrate. With 1 wt% MNPs, the morphology was maintained, but higher MNP amounts resulted in a nanostructure formed by cylinders perpendicular to the surface, despite some lamellae also were detected. This morphology evolution, in terms of AFM characterization, is shown in Figure 8. MNPs appeared to be well dispersed, without any agglomerations. The reason given by the authors for this evolution of the morphology was the following: as MNPs were selectively placed at PS domains (demonstrated by the elimination of the organic part by UV radiation), there was a reduction in their mobility. SQUID measurements were employed for the magnetic characterization of the nanocomposite films, showing that the magnetic properties were transferred to the nanocomposites.

In a second study [68], they modified the same maghemite MNPs with PMMA brushes via the grafting to technique to prepare nanocomposite thin films with PS-b-PMMA. Nanostructures both for the neat BCP and for the nanocomposites were obtained by SVA, with acetone employed as a selective solvent for PMMA blocks for 16 h, as was previously determined for obtaining a lamellar morphology for the neat BCP. They prepared nanocomposites with both silanized MNPs and those modified with brushes in order to compare the effect of the latter. The use of silanized MNPs resulted in the same lamellar morphology as that of the neat BCP, but with the presence of big agglomerates. Despite those agglomerates tending to gather at PMMA domains, as their size was bigger than that of the PMMA lamellae, they were not selectively placed there. With the purpose of selectively placing them in the PMMA domains, MNPs with PMMA brushes were employed. Even though the lamellar morphology was maintained for nanocomposites with 2 wt%, the morphology was not as regular as that of the neat BCP. No remarkable agglomerates were detected with MNPs at the PMMA domains. For nanocomposites of 5 wt%, the nanostructure started to change to a mixture of lamellae and perpendicular hexagonally packed cylinders. This seemed to indicate that modified MNPs were selectively placed at PMMA domains, altering the volume fraction among blocks and the equilibrium morphology. The authors degraded the organic part by UV radiation, showing that the MNPs were selectively placed. Moreover, SQUID measurements showed that the magnetic properties of nanoparticles were successfully transferred to the nanocomposite thin films. 

Finally, in a third study [69], they employed the grafting through technique to modify Fe_2_O_3_ NPs with PMMA or PS brushes for selective placement at the desired domains of a nanostructured poly(styrene-b-butadiene-b-styrene) (SBS) terpolymer. In order to analyze the effect of functionalization on the dispersion and selective placement of the NPs, nanocomposites were prepared by mixing an SBM copolymer with pristine, PS-, and PMMA-modified nanoparticles. Thin films of the neat BCP and nanocomposites (with 1, 2, and 5 wt% NPs) were prepared by casting SBM solutions in toluene onto silicon wafers. The morphology obtained was analyzed in terms of AFM. The neat BCP was nanostructured into a lamellar morphology, as found for ABA-type BCPs by other authors [4,70]. In this case, the lamellar structure showed an S–B–M–B sequence, with an average interlamellar distance of ~71 nm. For nanocomposites, independently of the modification with PMMA or PS, the lamellar morphology was maintained, with the MNPs appearing to be properly dispersed, without big aggregates. With the aim of analyzing the effect of brush type, nanocomposites with 5 wt% PMMA- and PS-modified MNPs were analyzed in detail. For PS-modified MNPs, domains corresponding to the PS block could be better distinguished in the AFM images, compared to the images obtained for nanocomposites with PMMA-modified MNPs. For nanocomposites with PMMA-modified MNPs, PS and PMMA domains tended to join together. The comparison of profile images showed that the PS and PMMA domains tended to swell with the addition of MNPs, while for the neat BCP, four domains could be distinguished. This could be a consequence of the placement of MNPs at the PS and PMMA domains, increasing the respective domain volume. The authors related the morphology differences detected between the neat BCP and the nanocomposite thin films to the effect of MNPs on system thermodynamics, altering the interaction among blocks. To show the effect of the surface modification of MNPs, they also prepared nanocomposite thin films under the same conditions as MNPs without any functionalization, concluding that even though the lamellar morphology was maintained, big aggregates were detected, with the dispersion not being as good as for modified MNPs. The transference of magnetic properties to the nanocomposites in all cases was demonstrated by magnetic characterization in terms of SQUID measurements.

## 4. Alignment with External Magnetic Field

In order to control the location and/or orientation of MNPs in nanocomposites with BCPs, different trials have been carried out, such as the employment of external shear or electric fields, in addition to the previously shown surface modification. In this way, some authors employed an external magnetic field for generating aligned BCP/MNP nanocomposites. Moreover, this magnetic field can also direct their neighboring BCP domains [29,31,36,37].

Hammond et al. [37] presented a method that can be used for the orientation of BCP cylinders, both in thin and in thicker films, and in some cases, even for bulk systems. They showed that rod-shaped NPs could nucleate coaxially oriented BCP cylinders. They analyzed a system based on PS-b-P2VP assembled into P2VP cylinders in a PS matrix and Fe_2_O_3_ MNPs. Due to the weak ferromagnetic nature of MNPs, they tended to orient towards their major axes perpendicular to the magnetic field. For this reason, nanocomposites were prepared as films with a thickness of 500 nm: MNPs laid in the film plane and perpendicular to the magnetic field, being oriented in the desired direction. In this way, a unique particle orientation was employed. Mixtures containing 0.05 wt% MNPs were cast onto carbon-coated epoxy or mica substrates and placed between two permanent magnets. The solvent was allowed to evaporate slowly. 

They concluded that NMPs effectively templated the orientation of nearby BCP cylinders. The application of a magnetic field during film casting led to a partial alignment of the MNPs, resulting in an overall alignment of the nanocomposite. Though the achieved alignment was modest, it can be considered a first step towards a complete alignment, opening a path for the generation of nanocomposites with proper combinations of anisotropic mechanical, magnetic, optical, and electrical properties, among others.

Yao et al. [36] also employed external magnetic fields to prepare nanocomposites based on a PS-b-PMMA copolymer and PS-coated Fe_2_O_3_ MNPs. By applying the magnetic field during the solvent casting procedure, they obtained nano- and microstructures by magnetic field-guided positioning of maghemite NPs. 

For low MNP concentrations, they were selectively placed at PS domains, but by increasing the MNP amount, they tended to aggregate, disrupting the morphology, as they were placed at the surface as wire-shaped stripes. The transference of magnetic properties to the nanocomposites was demonstrated, with the films showing superparamagnetic behavior and shape anisotropy, making them suitable for potential magnetic applications. They were able to generate highly oriented metal oxide wires with different widths and lengths by controlling the MNP amount and the intensity of the magnetic field. The change in the morphology of the BCP from symmetrical to asymmetrical was found to be due to the movement of MNPs within it, resulting in a highly anisotropic structure. The control of all those parameters could allow for the design of hybrid materials with many potential applications. 

**Figure 9:**
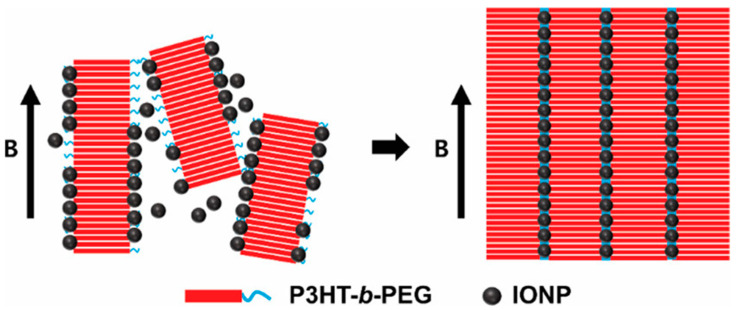
Schematic illustration of the self-assembly process of P3HT-b-PEG and iron oxide NPs in an external magnetic field B. (Reproduced with the permission of Ref. [31], copyright ACS 2022).

Park et al. [31] also employed a magnetic field to induce the self-assembly of poly(3-hexylthiopene-b-ethylene glycol) (P3HT-b-PEG) and iron oxide nanoparticles capped with oleic acid at the air–water interface. They claimed to generate, for the first time, thin films based on self-assembled BCP ordered at the nanoscale by the use of magnetic fields. The scheme of the process employed can be seen in Figure 9.

The relative amounts of MNPs and BCP employed assured the generation of uniform assembled structures, with the magnetic field intensity controlled by the distance between the magnets. They obtained nanoarrays of polymeric nanowires ordered in the long range and bridged with 1D nanoparticle chains. They pointed out the importance of MNP size. The generation of intermediate magnetic subunits composed by short BCP nanowires, which appeared to be decorated with MNPs, led to the self-assembly. Those subunits, as they claimed, could further assemble form nanoarrays ordered in the long range due to magnetic interactions generated by low-intensity magnetic fields. They could be employed in different potential applications in electronic, optoelectronic, or sensor devices, among others. 

Ren et al. [29] induced an order–order transition from hexagonally packed cylinders to long-range-oriented lamellae by the use of magnetic fields in nanocomposites formed by PS homopolymer, poly(styrene-b-ethylene oxide) (PS-b-PEO) copolymer, and a very small amount of Fe_3_O_4_ MNPs (0.07 vol%) modified on the surface with amino groups. They pointed out that this phase transition was induced by preforming a small-grain local domain comprising a mixture of magnetic field-assisted uniformly arranged NH_2_–Fe_3_O_4_ MNPs and a disordered PS/(PS-b-PEO) BCP melt as a nucleus, in which the interfacial tension at the junction points between PEO and PS blocks was significantly increased due to the enhancement in the block chain immiscibility so as to be able to induce the flat domain interface. This is in contrast with the phase transition kinetics usually found for neat BCPs. In this way, they found that a thermodynamically favored process drove the kinetic pathway, rather than a kinetics-dominated process, due to the alignment of MNPs occurring with higher particle amounts employed.

They also underlined the preferential orientation induced in both nanostructures: lamellar and hexagonally packed. Usually, when non-conductive or magnetically inactive BCPs are employed, a mechanical shear stress is responsible for the orientation, while the orientation can be induced by magnetic fields of low intensity by using very low amounts of MNPs. 

Table 1 shows the main advantages and disadvantages related to the procedures described for the development of BCP/MNP hybrid materials.

## 5. BCPs as Template Patterns for MNP Synthesis, Encapsulation, and Release

Several studies can be found in the literature on the use of BCPs as template patterns or nanoreactors for the generation of MNPs, for their encapsulation and release, for self-assembly inside BCP matrices, or for the generation of magnetic patterns based on metallopolymers or metal-containing BCPs, in some cases assisted by advanced techniques such as nanoimprint lithography (NIL) [38,39,40,41,42,43,44,45]. 

Regarding the employment of BCPs as a nanoreactor for MNP synthesis, the work of Ahmed et al. [41] must be cited. They employed the BCP nanoreactor route for the self-assembly of magnetic CoFe_2_O_4_ NPs in BCPs at room temperature. They employed diblock copolymers consisting of poly(norbornene) and poly(norbornene-dicarboxcylic acid) blocks (NOR-b-NORCOOH). The use of a template consisting of a self-assembled BCP made it possible to obtain MNPs at room temperature, as demonstrated by wide-angle X-ray diffraction and low-temperature Mossbauer-phase characterization. They processed the nanocomposites by wet chemical methods, obtaining a self-assembly of MNPs within the NORCOOH block at room temperature. The CoFe_2_O_4_ NPs were uniformly dispersed within the polymer matrix. They analyzed the magnetic properties of the obtained nanocomposite films, finding that they were superparamagnetic at room temperature and ferrimagnetic at 5 K.

Different studies can be found on the use of BCPs for the fabrication or encapsulation of MNPs in solution—in some cases, for their posterior release [38,39,40,42]. In this way, Hickey et al. [40] controlled the self-assembly structure of MNPs and amphiphilic block copolymers in solution by controlling MNP–solvent and MNP–BCP interactions. They employed poly(acrylic acid-b-styrene) (PAA-b-PS) copolymer and oleic acid-capped Fe_2_O_3_ MNPs.

By controlling solvent–MNP and BCP–MNP interactions, they obtained three different nanostructures: magneto-polymersomes densely packed with MNPs, magneto core–shells or core–shell-type BCP assemblies in which the MNPs appeared to be placed at the interface between the core and the shell, and magneto-micelles or BCP micelles in which the MNPs were homogeneously incorporated.

As MNP addition modified the volume ratio among the blocks, the self-assembly structure was altered, producing magneto-polymersomes instead of the classical magneto-micelles obtained using BCPs that self-assemble into micellar nanostructures. Moreover, polymers forming vesicles usually form magneto-micelles as a consequence of MNP solubilization inside BCP nanostructures. The MNP–BCP interaction also controlled the MNP arrangement. When N,N-dimethylformamide (DMF) was employed, in which PS was not well solvated, MNPs segregated from the PS, forming radial assemblies. On the contrary, by employing a good solvent for the PS and MNPs such as THF, they found a homogeneous distribution of MNPs into the BCP. They concluded that the morphology of the assembled BCP in which the MNPs were encapsulated greatly affected their magnetic relaxation properties.

Lecommandouxa et al. [39] also developed magnetic micelles and vesicles by self-assembly of polypeptide-based BCP and MNPs in water. Iron oxide NPs with magnetic response even to low-intensity magnetic fields were employed as the inorganic part, while the organic part was formed by poly(butadiene-b-glutamic acid) (PB-b-PGA) diblock copolymers able to self-assemble into micelles or vesicles. As the PGA block underwent a helix–coil transition, the formed peptosomes could act as stimuli-responsive capsules, varying their hydrodynamic diameter by as much as 50% in response to a pH change. 

They obtained 3D spherical micelles (micelles with a spherical shape) with a hydrophobic ferrofluid inside or hollow vesicles with a (2D, sheet-like structure) magnetic membrane, in which the MNPs appeared to be confined inside the layer of PB blocks. They concluded that the obtained soft magnetic objects could be used for drug delivery by the encapsulation of water-soluble species. The release of this content would be achieved by pH changes or by the use of magnetic fields, which could open the envelope. 

**Figure 10:**
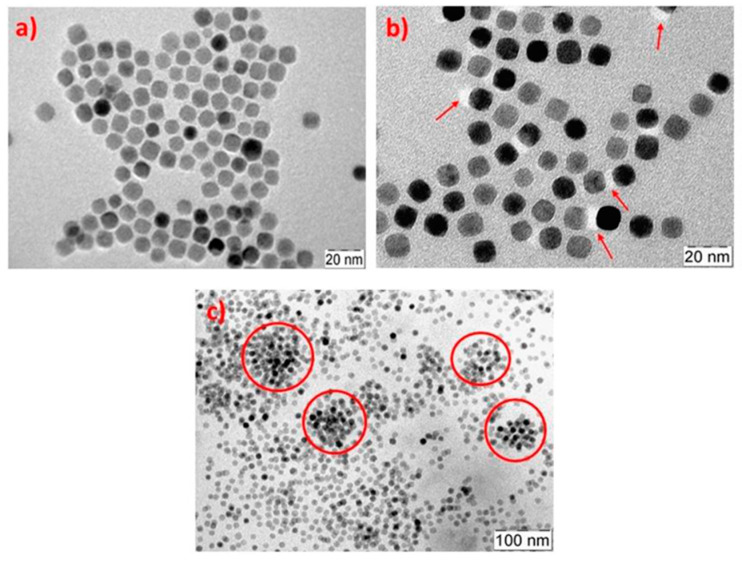
TEM images for (**a**) iron oxide NP-mixed, (**b**) PLMA-b-POEGMA/10% NP-mixed (red arrows indicate areas with a higher concentration of polymeric chains), and (**c**) PLMA-b-POEGMA/10% NP-mixed nanosystems after solvent evaporation (red circles indicate areas with large aggregation). (Reproduced with permission from Ref. [38]).

Skandalis et al. [38] employed micelle-forming poly(lauryl methacrylate-b-oligo ethylene glycol methacrylate) (PLMA-b-POEGMA) amphiphilic block copolymers for the encapsulation of magnetic iron oxide nanoparticles with water as the solvent. The self-assembly of BCPs in water resulted in micelles and could contain MNPs of up to 10 wt% in their PLMA cores, forming mixed nanostructures, as shown in Figure 10. Comparing the size and distribution of micelles for the BCP and the nanocomposite, they were significantly broader for the latter. By magnetophoresis, the authors demonstrated that the solutions maintained the magnetic properties, as MNPs continued being magnetic after the encapsulation. They also checked that even after the encapsulation of a drug (indomethacin) together with the MNPs, the magnetic properties were maintained. 

Morcrette et al. [42] fabricated NPs (with a very narrow size dispersion of around 7%) released in solution with the use of BCP as a patterned template. They employed a combined “top-down” and “bottom-up” approach with the use of a self-assembled PS-PMMA BCP formed on a sacrificial layer. The self-assembly of PS-b-PMMA required an intermediate layer composed of a “random” PS-r-PMMA copolymer with the same composition as the BCP. The reason for employing this sacrificial layer was substrate surface neutralization, which is necessary for obtaining a vertical cylinder-type morphology rather than a horizontal one, which is obtained without the neutralization of the surface. A cross-linking process, induced by a thermal annealing performed after the spin coating, allowed for the grafting of the layer to the sacrificial germanium substrate (previously deposited onto a silicon substrate, which is important for the subsequent lift-off of NPs). Then, the PS-b-PMMA was spin coated and thermally annealed to provide mobility to the polymer chains for self-assembly. PMMA cylinders were removed by employing acetic acid, obtaining a hexagonal template of holes in the PS matrix. After that, they removed the neutral copolymer layer for the deposition of the magnetic material on the cited template by evaporation or DC sputtering. Finally, after removing the PS template by plasma exposure, a packed hexagonal array of NPs was obtained. The method was successfully employed for the fabrication of different NPs such as gold-coated Ni, FeNi, and Fe_2_O_3_. The use of the germanium oxide sacrificial layer, compatible with BCP mask fabrication, made the release of MNPs in solution possible. In addition to the main potential applications in imaging (MRI, MPI), as anisotropic MNPs with a small size could be obtained, the generated materials could also be employed in other biomedical applications, such as treatments for cancer cell destruction. 

**Figure 11:**
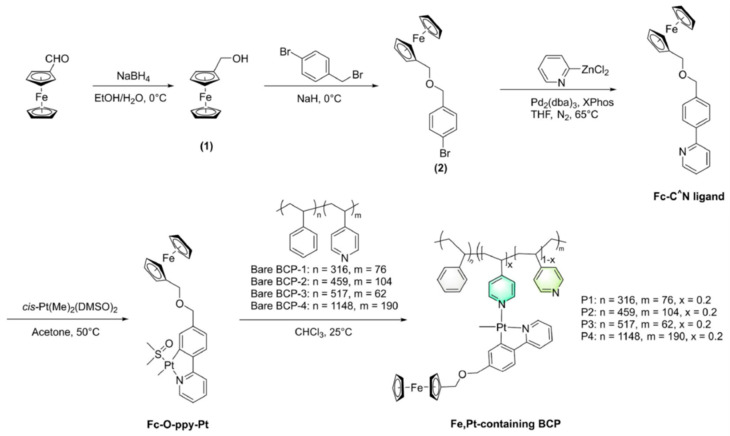
Synthetic route for obtaining Fe, Pt-containing BCPs (reproduced with permission from Ref. [44], copyright Springer Nature 2025).

Recently, Zhang et al. [44] also employed self-assembled BCPs (in this case, bimetallic or metal-containing BCPs) to obtain magnetic patterns of Fe, Pt NPs, assisted by lithography nanoimprint (NIL). They synthesized four new Fe, Pt-containing BCPs, named P1–P4, selecting a PS-b-P4VP BCP (with the possibility of self-assembly, containing pyridine groups as effective coordination sites for metallic ions) to be employed as a precursor for the generation of monodisperse FePt MNPs without using any surfactant. The post-coordination of the BCP allowed those bimetallic BCPs to be obtained directly, keeping the atomic ratio of Fe to Pt identical. The synthesis procedure is shown in Figure 11.

Among these four bimetallic copolymers, P3 and P4 were the most easily assembled, especially P3, which generated spherical assemblies by solvent vapor annealing with dioxane. For this reason, they chose P3 for the nanoimprint lithography-assisted method, which is shown in detail in the experimental section of their paper [44].

They used the nanoimprint lithography-assisted self-assembly method to obtain large-scale regular patterns, leading to the consequent self-assembly of the BCP into nanodomain arrays without any defect in the previously defined features. After the thermal decomposition (in situ pyrolisis), the patterned metal-containing copolymer was completely transformed into Fe, Pt-based magnetic patterns. They concluded that their materials could present potential applications in ultra-high-density data storage systems.

Metal-containing copolymers were also employed by other authors for the generation of MNPs. Al-Badri et al. [43] synthesized Co-containing novel BCPs by living ring-opening metathesis polymerization. Those novel BCPs could perform microphase separation and, after a heat treatment, presented ferromagnetic properties at room temperature. As the employment of the parent homopolymer generated only paramagnetic materials, the nanostructuring was shown to be a key parameter. 

They claimed that the origin of the ferromagnetic properties shown at room temperature (room-temperature ferromagnetic materials (RTFM)) came from the nanostructured confinement of Co within the one-dimensional cylinders of the BCP morphology. The fact that the particles were too small (~5 nm) to present ferromagnetism at room temperature and, moreover, that they were present in both the BCP and the homopolymer, with only the former generating a ferromagnetic material, supported the hypothesis. They further demonstrated the cited hypothesis by synthesizing the BCPs with different morphologies (cylindrical, lamellar, and inverted cylindrical phases). All the nanostructured morphologies led to RFTM, in contrast to the homopolymer, which did not present any nanostructure and did not lead to any RTFM. Moreover, the cylindrical BCP, which was the most strongly confined, presented the best ferromagnetic properties, followed by the inverted cylindrical morphology.

Yiu et al. [45] also employed bimetallic BCP precursors for the generation of FePt MNPs. They incorporated a complex containing Fe and Pt centers into the coordinating block of four different PS-b-P4VP (named P1–P4) copolymers for the synthesis of bimetallic BCPs. They analyzed both thin-film and bulk self-assembly, finding that the spherical and cylindrical morphologies observed corresponded with those of non-metallic BCP, but, of course, in the case of metallic BCPs, the phase-separated cylindrical or spherical nanodomains contained FePt NPs. Once the organic part was pyrolized (at 800 °C in an inert atmosphere), the pyrolisis products of those bimetallic BCPs were analyzed, and they found that the formed FePt NPs presented an fct phase, with average sizes of between 4 and 8 nm. They demonstrated the importance of nanostructuring BCPs for the synthesis of ferromagnetic FePt NPs with a smaller average size with a Fe/Pt stoichiometric ratio close to 1:1 by comparing results obtained with the corresponding P4VP homopolymer. They concluded by pointing out that metallo-BCP lithography was an effective procedure for obtaining FePt NPs from a bimetallic source without the use of any surfactant. 

## 6. Conclusions

This review work shows the implementation of nanostructured BCPs and MNPs, combined in several ways and prepared by several methods, to obtain interesting materials with potential applications in different fields such as biomedicine, optoelectronics, or data storage devices. The main conclusions that can be extracted from the use of different procedures and their success are the following.

The first procedure for their preparation is the nanostructuring of BCPs in the presence of previously synthesized NPs by modifying their surface (with surfactants of polymeric brushes) to increase their compatibility and selectively place them at nanodomains or by employing external magnetic fields for orientation.

By employing surfactants, the degree of interaction between MNPs and one of the blocks determines the dispersion and selective NP placement, as well as the effect on BCP morphology. In any case, the magnetic properties are effectively transferred to the nanocomposite.

The method consisting of the grafting of polymeric brushes has been shown to be more effective than the use of surfactants. Brushes can be grafted to the surface of NPs by three main methods, known as grafting from, grafting through, and grafting to. 

By comparing the three methods, the last one has been found to be the least effective for NP confinement. The other two methods are effective, but the dispersion and placement strongly depends on the grafting density and the molar mass of the brushes. The selective placement of NPs at certain domains can affect the morphology of the BCP, which can also be disrupted for higher NP contents if they tend to form aggregates. 

In all cases, the magnetic properties are effectively transferred to the nanocomposites, resulting in materials with potential optoelectronic applications. 

The use of external magnetic fields for the orientation of NPs and even that of the nearby domains has been employed with success, strongly depending on the amount of NPs and the magnetic field intensity. By properly controlling both parameters, long-range-ordered nanoarrays of polymeric nanowires bridged with NP chains can be obtained.

The second procedure, consisting of the in situ synthesis of NPs inside the nanodomains by a reduction in the respective metallic ions or by employing metal-containing BCPs for the generation of MNPs patterns or arrays, has been successfully applied to obtain selective dispersion and the transference of magnetic properties.

Finally, the synthesis of metallopolymers or metal-containing BCPs to generate magnetic patterns of different MNPs has also been explored by different authors, in some cases assisted by the use of advanced techniques such as nanoimprint lithography. Obtaining MNPs with better size control and properties by using nanostructured BCPs instead of homopolymers has shown the importance of nanostructuring and confinement for a more accurate generation of MNPs with ferromagnetic properties.

## Figures and Tables

**Table 1:** Main advantages and disadvantages of the procedures employed for hybrid material preparation.

Process	Advantages	Disadvantages
Surfactant	Easy to perform Many surfactants available.	NPs tend to form aggregates Less efective than brushes for selective placement.
Grafting to	Acceptable grafting densities. Control of molecular weight of grafted chains. Success in the proper dispersion and selective placement of modified NPs into desired domains.	Limitation on the choice of functional groups available. The thickness of the film, and accordingly the number of functional groups per surface area that can be obtained. Due to the inability of large polymer chains to diffuse to the reactive surface sites, they are sterically hindered by the surrounding bounded chains.
Grafting from	With the development of controlled living radical polymerization methods such as ATRP or RAFT some issues can be improved.	Poor control of molecular weight, polydispersity, end functionality, chain architecture and composition. Stringent conditions and the small number of monomers that can be polymerized in ionic living polymerization method.
Grafting through	Widely applied, even in industrial applications mostly for adhesion promotion. Success in the proper dispersion and selective placement of modified NPs into desired domains.	This approach has not been extensively explored and the details of the mechanism are not well understood. Poor grafting density control.
Magnetic field	Successfully applied even with very small NP amounts. Orientation of both MNPs and surrounding BCP domains. Very promissing procedure for biomedical applications.	Not easy for transference to industrial processes. Higher energy comsuption than other processes. Has to be improved and further investigated.

## Data Availability

The data presented are available on request from the corresponding author.
